# Influence of Vinegar and Wine Processing on the Alkaloid Content and Composition of the Traditional Chinese Medicine Corydalis Rhizoma (Yanhusuo)

**DOI:** 10.3390/molecules190811487

**Published:** 2014-08-04

**Authors:** Hongwei Wu, Katharina Waldbauer, Liying Tang, Lianwu Xie, Ruxandra McKinnon, Martin Zehl, Hongjun Yang, Haiyu Xu, Brigitte Kopp

**Affiliations:** 1 Institute of Chinese Materia Medica, China Academy of Chinese Medical Sciences, Dong Nei Nan Xiao Jie 16, Beijing 100700, China; 2 Department of Pharmacognosy, University of Vienna, Althanstrasse 14, Vienna A-1090, Austria; 3 College of Sciences, Central South University of Forestry and Technology, 498 South Shaoshan Road, Changsha 410004, China

**Keywords:** Corydalis Rhizoma, Yanhusuo, alkaloids, processing, traditional Chinese medicine

## Abstract

Corydalis Rhizoma is the dried tuber of *Corydalis yanhusuo* W.T. Wang which is used in traditional Chinese medicine for pain relief and blood activation. Before being used in the clinics, *C. yanhusuo* is traditionally processed through dry-frying or frying with vinegar, wine or salt. In this study, eleven alkaloids from Corydalis Rhizoma, namely protopine (**1**), α-allocryptopine (**2**), tetrahydrocolumbamine (**3**), coptisine (**4**), palmatine (**5**), berberine (**6**), dehydrocorydaline (**7**), d,l-tetrahydropalmatine (**8**), tetrahydroberberine (**9**), corydaline (**10**) and tetrahydrocoptisine (**11**) were simultaneously quantified using a newly developed high performance liquid chromatography-diode array detector (HPLC-DAD) method. The influence of vinegar and wine processing on the content of the main alkaloids of Corydalis Rhizoma was investigated. For this purpose, two common formulations with clinical application, namely the water decoction of Corydalis Rhizoma and its formula Jin Ling Zi San (combination of Corydalis Rhizoma and Toosendan Fructus) were studied. In the two water decoctions, wine and vinegar processing increased the amount of tertiary alkaloids. The differences were more pronounced for Jin Ling Zi San, in which case the content of all tertiary alkaloids (compounds **1**, **2**, **3**, **8**, **9**, **10**, **11**) was increased by wine processing.

## 1. Introduction

Corydalis Rhizoma, known as Yanhusuo in China, is prepared from the dried tuber of *Corydalis yanhusuo* W.T. Wang. In traditional Chinese medicine *C. yanhusuo* is believed to have the function of activating blood, moving “Qi” (vital energy) and relieving pain. Therefore, Corydalis Rhizoma is used in clinics as a herbal medicine for the treatment of chest impediments, heart pain, amenorrhea and dysmenorrhea, postpartum stasis and obstruction [1].

To date, the chemical constituents of Yanhusuo have been isolated and identified as tertiary and quaternary alkaloids which are responsible for the biological activities of the crude drug [2]. Alkaloids such as tetrahydropalmatine exert their pharmaceutical effect by interfering with neurotransmitters in the central nervous system [3,4,5,6]. Other biological activities of the alkaloids or crude extract from Corydalis Rhizoma were also found, such as anti-tumor activity [7,8,9], anti-inflammatory and analgesic activity [10,11,12], hepatoprotective effects [13], and antiplasmodial activity [14]. Several high performance liquid chromatography (HPLC) methods have been reported for the analysis of alkaloids in *C. yanhusuo.* Quantification is typically performed using ultraviolet (UV) detection while diode array detector (DAD) and mass spectrometry are often employed for the identification [15,16].

Prior to their usage in clinics, crude drugs are subjected to traditional Chinese processing techniques (PaoZhi) such as cleaning, cutting and stir-baking with various excipients. Only processed slices are used for decoctions in clinics and as raw material for pharmaceutical manufacturing. In the Materia Medica, Bensky *et al.* [17] mention four methods of preparation for Corydalis Rhizoma, namely dry-, vinegar-, wine- and salt-frying, and point out that there are large differences in the alkaloid content between the processed products. As first documented in Lei’s Treatise on Processing of Drugs (Leigong PaoZhi Lun) in the Tang Dynasty of China (618–907 AD) [18], Corydalis Rhizoma has been used mainly in vinegar and wine processed form. The preparation deepens the external color of the tubers and is believed to promote blood activation and pain relief. The difference between vinegar and wine processing is given by the processing adjuvants, yellow wine and rice vinegar, respectively. The function of yellow wine is to improve the pain relief effect, while rice vinegar increases the efficacy of the herbal drug [17]. Previous reports showed that vinegar processing can improve the analgesic effect of Yanhusuo and can also change the pharmacokinetics of the main alkaloids [19,20]. With regard to the associated changes in composition, only a few of the main compounds of Yanhusuo have been studied*.* For example, vinegar processing was shown to cause an increase in tetrahydropalmatine and a decrease in berberine and protopine [21,22].

In the clinic, the plant is often used as a water decoction of Corydalis Rhizoma alone or in combination with fruits of *Melia toosendan* Sieb. et Zucc. (Toosendan Fructus). The latter formulation is known under the name of Jin Ling Zi San (JLZS) and has been used to relieve chest impediments caused by “liver fire”, according to the medicinal book Suwen Bingji Qiyi Baoming Ji from the Jing Dynasty of China (1186 AD) [23]. Bensky *et al.* [17] report that Toosendan Fructus is used to alleviate pain in the flank and upper right quadrant and acts synergistically with Corydalis Rhizoma. The main constituents of Toosendan Fructus have been identified as tetracyclic triterpenes, polysaccharides and phenolic acids [24].

Despite the frequent use of Corydalis Rhizoma and JLZS in clinics, there are few reports concerning the influence of processing on their alkaloid composition. Hence, a thorough analysis of the influence of wine and vinegar processing on the alkaloid content of Rhizoma Corydalis and its common formulations is missing.

**Figure 1:**
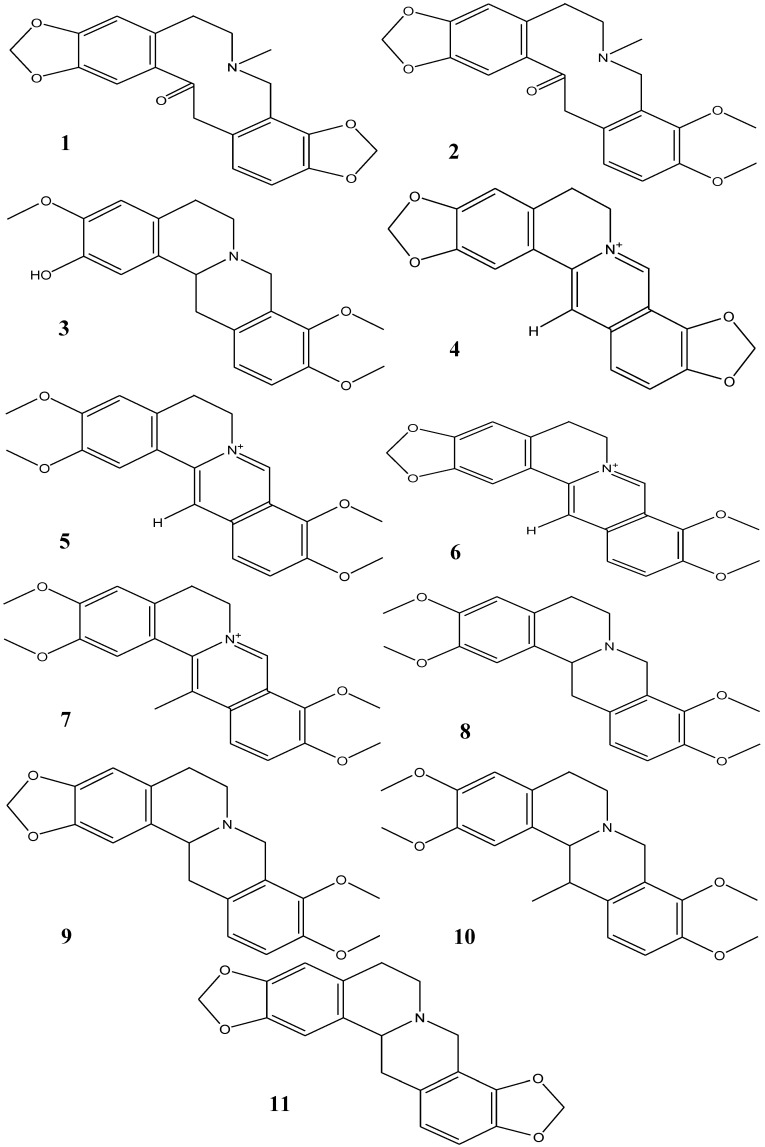
Chemical structures of the main alkaloids detected in Corydalis Rhizoma. Protopine (**1**), α-allocryptopine (**2**), tetrahydrocolumbamine (**3**), coptisine (**4**), palmatine (**5**), berberine (**6**), dehydrocorydaline (**7**), d,l-tetrahydropalmatine (**8**), tetrahydroberberine (**9**), corydaline (**10**), tetrahydrocoptisine (**11**).

In this study, a HPLC method was developed for the simultaneous analysis of 11 main alkaloids from the tubers of Corydalis Rhizoma*:* protopine (**1**), α-allocryptopine (**2**), tetrahydrocolumbamine (**3**), coptisine (**4**), palmatine (**5**), berberine (**6**), dehydrocorydaline (**7**), d,l-tetrahydropalmatine (**8**), tetrahydroberberine (**9**), corydaline (**10**) and tetrahydrocoptisine (**11**) (Figure 1). The method was then employed to determine the alkaloid content and composition of the methanol extract of Corydalis Rhizoma. Using the methanol extract as a point of comparison, the influence of vinegar and wine processing on the alkaloid content and composition was evaluated for two common formulations with clinical application: the water decoction of Corydalis Rhizoma and its formula JLZS. 

## 2. Results and Discussion

### 2.1. Method Development and Validation

For the simultaneous determination of the main alkaloids of Yanhusuo, a HPLC-DAD method was developed and validated. Three columns-Agilent TC-C18, Phenomenex Gemini C18 and Dikma Diamonsil II C18-were tested for the separation process, of which the Phenomenex Gemini C18 one gave the best chromatographic resolution. The pH value of the mobile phase influenced the shapes of the peaks and also changed the elution order of the detected alkaloids. A solution of 0.2% acetic acid adjusted with triethylamine to pH 5.0 was found to give symmetric peak shapes. To avoid errors caused by batch to batch variation, all samples of unprocessed and processed Corydalis Rhizoma originated from the same batch. Powdered plant material was used for methanol extraction. Slices of unprocessed and processed Corydalis Rhizoma were used for the water decoctions. The methanol extract served as a standard for comparison of the alkaloid content and composition with the water decoctions used in traditional Chinese medicine. The methanol was evaporated at 40 °C and the water decoctions were dried by lyophilization to avoid degradation of the alkaloids caused by high temperature.

**Figure 2:**
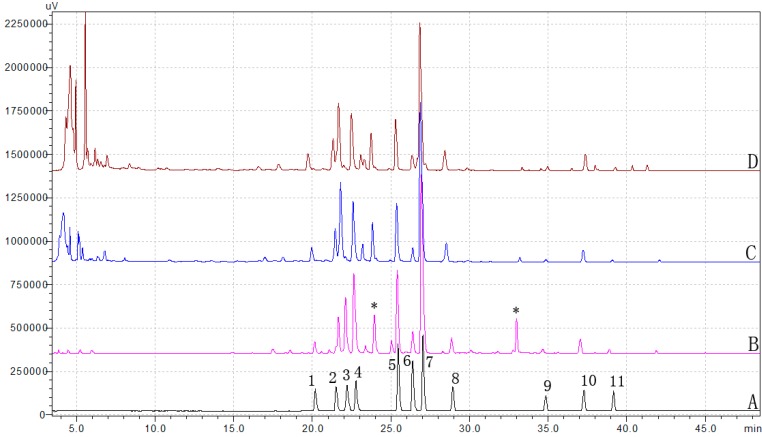
Representative HPLC chromatograms of Corydalis Rhizoma samples and reference compounds. Mixture of reference compounds (**A**); methanol extract of Corydalis Rhizoma (**B**); water decoction of Corydalis Rhizoma (**C**); water decoction of JLZS (**D**). Protopine (**1**), α-allocryptopine (**2**), tetrahydrocolumbamine (**3**), coptisine (**4**), palmatine (**5**), berberine (**6**), dehydrocorydaline (**7**), DL-tetrahydropalmatine (**8**), tetrahydroberberine (**9**), corydaline (**10**), tetrahydrocoptisine(**11**); two additional abundant compounds of **B** which were not quantified (see main text) are indicated with an asterisk (*).

Figure 2 shows that all 11 alkaloids were successfully separated by the method. In order to identify the alkaloids in the methanol extract of Corydalis Rhizoma, the samples and reference compounds were further analyzed by liquid chromatography-mass spectrometry (LC-MS).

According to the retention time, UV and mass spectra, the presence of each of the 11 alkaloids in the samples was confirmed (Table 1). Two additional abundant peaks were detected in the methanol extract of Corydalis Rhizoma (indicated with an asterisk in Figure 2). The compound eluting at ~24 min yielded an M^+^ ion at *m/z* 352.2 Da and an UV spectrum typical for the quaternary protoberberine-type alkaloids. By comparison of the MS^n^ spectra and elution order to literature data, this compound can be assigned as pseudopalmatine [25].

The constituent that eluted after ~33 min yielded an [M+H]^+^ or M^+^ ion at *m/z* 352.1 Da, but could not be identified based on the available data. However, the abundance of this compound in the water decoctions, which are more relevant to the way Yanhusuo is used in the clinic, is very low.

**Table 1:** HPLC-DAD and LC-MS data for alkaloids of Corydalis Rhizoma.

Compound	Retention Time (min) ^a^	Precursor Ion (*m/z*)	Main MS^2^ Fragment Ions (>10% rel. int.) (*m/z*)	λ_max_ (nm)
**1**	20.2	354.1 [M+H]^+^	336.4 (21), 323.3 (11), 275.3 (33), 247.3 (16), 206.3 (11), 189.3 (67), 188.3 (100), 149.3 (79), 119.4 (17)	211, 239, 288
**2**	21.5	370.1 [M+H]^+^	352.4 (42), 291.3 (12), 290.3 (25), 206.3 (12), 188.3 (100)	209, 229, 284
**3**	22.2	342.2 [M+H]^+^	178.3 (100)	211, 226, 282
**4**	22.8	320.1 M^+^	292.3 (100), 260.8 (13)	228, 238, 266, 358, 460
**5**	25.4	352.2 M^+^	337.4 (100), 336.4 (55), 308.3 (64)	226, 265, 272, 345, 423
**6**	26.4	336.1 M^+^	321.3 (100), 320.3 (48), 292.3 (47)	230, 264, 347, 427
**7**	27.0	366.2 M^+^	351.4 (100), 350.4 (76), 322.4 (51)	230, 263, 336, 417
**8**	28.9	356.2 [M+H]^+^	192.3 (100), 165.3 (23)	211, 230, 281
**9**	34.8	340.1 [M+H]^+^	176.3 (100)	208, 231, 285
**10**	37.3	370.2 [M+H]^+^	218.3 (12), 205.3 (16), 192.3 (93), 179.3 (16), 165.3 (100), 151.3 (14), 150.3 (39), 136.3 (12), 135.4 (12)	211, 229, 282
**11**	39.2	324.1 [M+H]^+^	176.3 (100), 149.3 (54)	201, 237, 289

^a^ The given retention times refer to the HPLC-DAD method (see 3.5.). The compounds eluted 2–3 min earlier on the LC-MS system (see Section 3.6).

The linearity of the detector response at 280 nm for each compound was examined using a dilution series of a mixture of reference compounds. A calibration curve was constructed using the linear regression of the peak area *versus* the concentration of the analyte. The equations, linear ranges, limits of detection (LOD) and quantification (LOQ) for the 11 compounds are summarized in Table 2. The intra- and inter-day relative standard deviations (RSD) were 0.92%–2.19% and 2.18%–4.91%, respectively (Table 3).

**Table 2:** Calibration data for the alkaloids of Corydalis Rhizoma.

Compound	Regression Equation	r^2^	Linear Range (μg)	LOQ (μg)	LOD (μg)
1	*y* = 1062369*x* + 14016	0.9997	4.120–0.008	0.008	0.004
2	*y* = 799236*x* + 12725	0.9998	6.260–0.012	0.012	0.006
3	*y* = 838091*x* + 26101	0.9994	6.600–0.013	0.013	0.006
4	*y* = 2514102*x* + 11571	0.9998	2.630–0.005	0.005	0.003
5	*y* = 3704272*x* + 51320	0.9997	3.400–0.007	0.007	0.003
6	*y* = 2483438*x* + 31925	0.9997	4.040–0.008	0.008	0.004
7	*y* = 3483721*x* + 57730	0.9997	4.100–0.008	0.008	0.004
8	*y* = 931781*x* + 18150	0.9997	5.200–0.010	0.010	0.005
9	*y* = 1029317*x* + 6872	0.9998	3.040–0.006	0.006	0.003
10	*y* = 809393*x* + 23046	0.9996	5.500–0.011	0.011	0.005
11	*y* = 665716*x* + 14423	0.9997	5.600–0.011	0.011	0.005

Peak area (*y*); amount of injected compound (*x)*, μg; Limits of quantification (LOQ); Limits of detection (LOD).

**Table 3:** Precision, repeatability and recovery data for alkaloids of Corydalis Rhizoma.

Compound	Precision				Repeatability (*n* = 6)		Recovery (*n* = 6)	
	Intra-Day (*n* = 3)		Inter-Day (*n* = 9)		Mean (mg/g)	RSD (%)	Mean (%)	RSD (%)
	Mean (mg/mL)	RSD (%)	Mean (mg/mL)	RSD (%)				
**1**	0.116	1.26	0.121	3.35	0.31	0.86	99.7	4.01
**2**	0.169	1.50	0.169	2.71	1.19	1.70	102.3	1.50
**3**	0.173	2.17	0.181	4.91	1.87	3.38	97.1	1.53
**4**	0.057	2.19	0.058	3.20	0.85	0.59	94.1	2.92
**5**	0.085	1.05	0.089	3.54	0.55	2.02	96.9	3.91
**6**	0.135	1.06	0.140	3.31	0.29	2.63	97.5	2.77
**7**	0.131	0.95	0.137	3.69	2.09	1.78	100.1	3.71
**8**	0.137	0.92	0.143	3.36	0.41	1.96	97.2	1.80
**9**	0.082	1.76	0.083	2.18	0.12	1.44	99.4	2.21
**10**	0.145	1.11	0.151	3.26	0.51	2.02	97.3	3.18
**11**	0.148	1.04	0.153	2.94	0.16	2.89	99.6	4.35

To confirm the repeatability, six parallel methanol extracted samples of Corydalis Rhizoma were analyzed. The RSD of the 11 alkaloids detected were 0.59%–3.38%. The measured recoveries of compounds **1**–**11** ranged from 94.1% to 102.3% with an RSD between 1.5% and 4.3%. Due to the varying size of prepared plant material, the RSD for alkaloids detected in the two water decoctions (obtained from tuber slices) was higher than the RSD provided by the methanol extract (obtained from powdered tuber). 

### 2.2. Influence of Vinegar and Wine Processing on the Total Alkaloid Content of Corydalis Rhizoma

**Figure 3:**
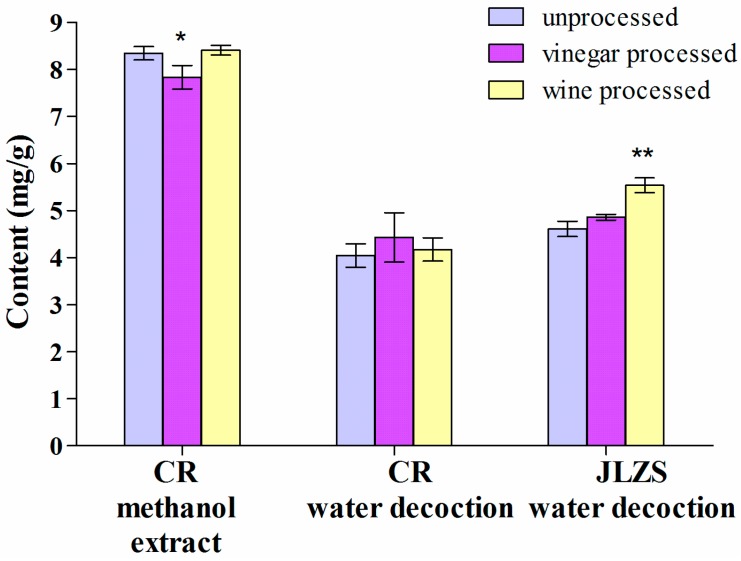
Alkaloid content in processed and unprocessed Corydalis Rhizoma. Corydalis Rhizoma (CR), Jin Ling Zi San (JLZS). Values are mean ± SD, *n* = 3, RSD < 6%. ** *p* < 0.01, * *p* < 0.05 of processed against unprocessed Corydalis Rhizoma.

Analysis of the total alkaloid content showed significantly lower values for vinegar processed *versus* unprocessed or wine processed Corydalis Rhizoma in the methanol extract (Figure 3, Table 4). Of the two water decoctions, only JLZS showed significant changes in the alkaloid content which increased when using wine processed drug. Hence, the results are consistent with the traditional experience of using wine processed Corydalis Rhizoma for the JLZS decoction. The results also indicate that the alkaloid content in the water decoction is influenced not only by processing, but also by the plant material added to Corydalis Rhizoma.

Comparison of the chromatograms of processed and unprocessed Yanhusuo (not shown) revealed no obvious new peaks, which indicated that processing mainly caused quantitative changes. Usage of pulverized material (processed and unprocessed) led to an increase in the alkaloid content in the methanol extract when compared to the water decoctions obtained from tuber slices. 

According to Bensky *et al.* [17], of the total alkaloids (100%) the availability is as follows: decoction of unprocessed drug (25.06%), decoction of vinegar processed drug (49.33%), decoction of wine processed drug (22.66%) [17]. However, our study yielded different values. When the total alkaloids in the methanol extract were considered 100%, the alkaloid availability in the decoction of unprocessed drug was 48.44%, of vinegar processed 56.44% and of wine processed 49.58%.

**Table 4:** Alkaloid content of unprocessed and processed Corydalis Rhizoma.

Compound		1	2	3	4	5	6	7	8	9	10	11	Total amount
**Methanolextract (mg/g)**	**UP**	0.310 ± 0.003	1.191 ± 0.020	1.866 ± 0.063	0.851 ± 0.005	0.548 ± 0.011	0.286 ± 0.008	2.093 ± 0.037	0.406 ± 0.008	0.120 ± 0.002	0.511 ± 0.010	0.162 ± 0.005	8.342 ± 0.141
	**VP**	0.301 ± 0.010	1.133 ± 0.040	1.826 ± 0.036	0.752 ± 0.030 **	0.504 ± 0.010 **	0.248 ± 0.014 *	1.889 ± 0.087 *	0.397 ± 0.015	0.122 ± 0.003	0.513 ± 0.006	0.157 ± 0.004	7.833 ± 0.250 *
	**WP**	0.296 ± 0.007 *	0.997 ± 0.007 **	2.268 ± 0.035 **	0.724 ± 0.005 **	0.381 ± 0.004 **	0.203 ± 0.003 **	1.614 ± 0.025 **	0.701 ± 0.016 **	0.169 ± 0.002 **	0.806 ± 0.015 **	0.248 ± 0.002 **	8.406 ± 0.106
**Water decoction (mg/g)**	**UP**	0.216 ± 0.002	0.559 ± 0.013	1.340 ± 0.123	0.290 ± 0.019	0.180 ± 0.013	0.084 ± 0.006	0.800 ± 0.059	0.321 ± 0.006	0.032 ± 0.003	0.189 ± 0.012	0.033 ± 0.004	4.040 ± 0.251
	**VP**	0.249 ± 0.024	0.644 ± 0.072	1.473 ± 0.179	0.300 ± 0.037	0.204 ± 0.017	0.092 ± 0.002	0.851 ± 0.114	0.381 ± 0.023 **	0.040 ± 0.004	0.233 ± 0.018 *	0.044 ± 0.006	4.430 ± 0.526
	**WP**	0.241 ± 0.010 *	0.585 ± 0.032	1.379 ± 0.050	0.281 ± 0.023	0.177 ± 0.014	0.078 ± 0.005	0.797 ± 0.063	0.339 ± 0.026	0.036 ± 0.003	0.224 ± 0.025	0.036 ± 0.003	4.170 ± 0.245
**Jin Ling Zi Sandecoction(mg/g)**	**UP**	0.260 ± 0.021	0.599 ± 0.030	1.448 ± 0.077	0.344 ± 0.032	0.199 ± 0.014	0.102 ± 0.006	0.859 ± 0.050	0.434 ± 0.002	0.053 ± 0.000	0.301 ± 0.026	0.056 ± 0.002	4.610 ± 0.164
	**VP**	0.267 ± 0.023	0.596 ± 0.030	1.632 ± 0.015 *	0.316 ± 0.027	0.189 ± 0.011	0.092 ± 0.006	0.832 ± 0.044	0.415 ± 0.020	0.057 ± 0.001	0.326 ± 0.003	0.066 ± 0.006	4.860 ± 0.064
	**WP**	0.305 ± 0.023 *	0.658 ± 0.026 *	1.878 ± 0.008 **	0.344 ± 0.014	0.212 ± 0.011	0.103 ± 0.006	0.924 ± 0.029	0.523 ± 0.025 **	0.069 ± 0.005 **	0.427 ± 0.006 **	0.091 ± 0.005 **	5.530 ± 0.158 **

Unprocessed (UP), vinegar processed (VP), wine processed (WP); Values are mean ± SD, *n* = 3; ** *p* < 0.01, * *p* < 0.05 of processed versus unprocessed.

### 2.3. Influence of Vinegar and Wine Processing on the Alkaloid Content of the Methanol Extract of Corydalis Rhizoma

**Figure 4:**
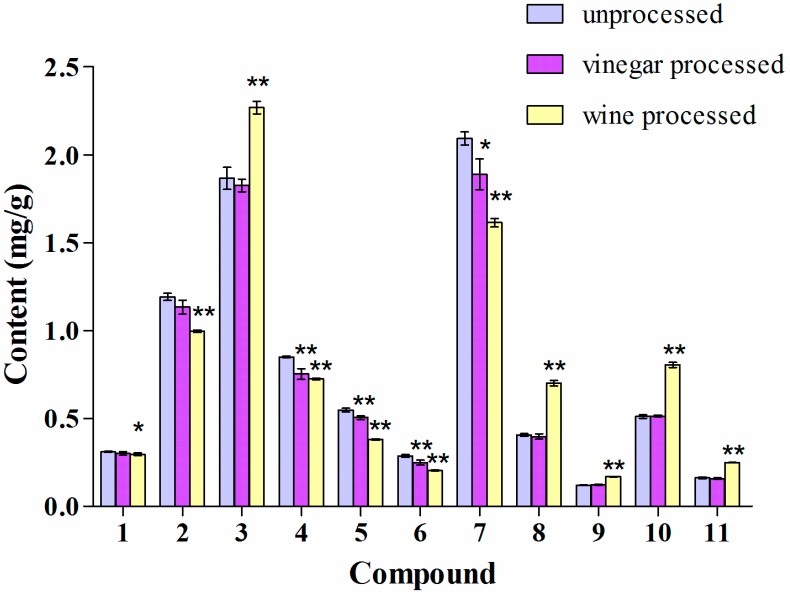
Alkaloid content of the methanol extract of Corydalis Rhizoma. Values are mean ± SD, *n* = 3, RSD < 4%. ** *p* < 0.01, * *p* < 0.05 of processed against unprocessed Corydalis Rhizoma.

As shown in Figure 4, vinegar and wine processing both caused a significant decrease in the content of the quaternary alkaloids **4**, **5**, **6**, and **7** when compared to unprocessed Yanhusuo. This could be due to the weak basic properties of quaternary alkaloids that are known to form unstable salts in acidic medium [26]. In addition, wine processing also caused changes in the amount of tertiary alkaloids as follows: the content of compounds **1**, and **2** decreased and that of **3**, **8**, **9**, **10**, and **11** increased.

### 2.4. Influence of Vinegar and Wine Processing on the Alkaloid Content of the Water Decoctions of Corydalis Rhizoma and JLZS

**Figure 5:**
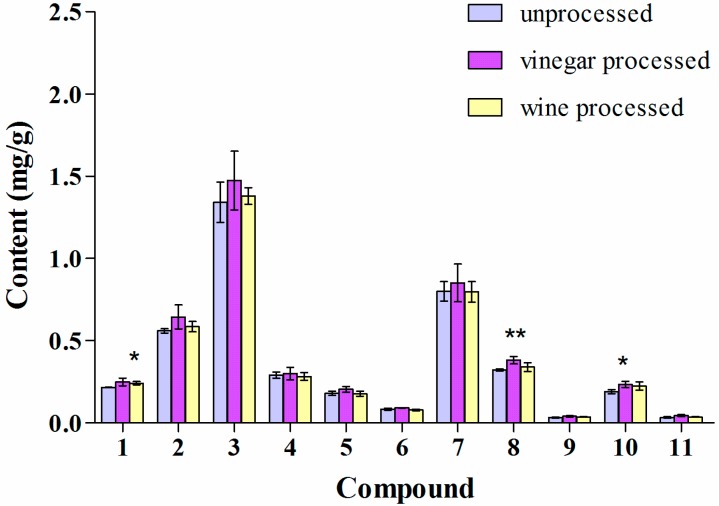
Alkaloid content of the water decoction of Corydalis Rhizoma. Values are mean ± SD, *n* = 3, RSD < 13%. ** *p* < 0.01, * *p* < 0.05 of processed against unprocessed Corydalis Rhizoma.

**Figure 6:**
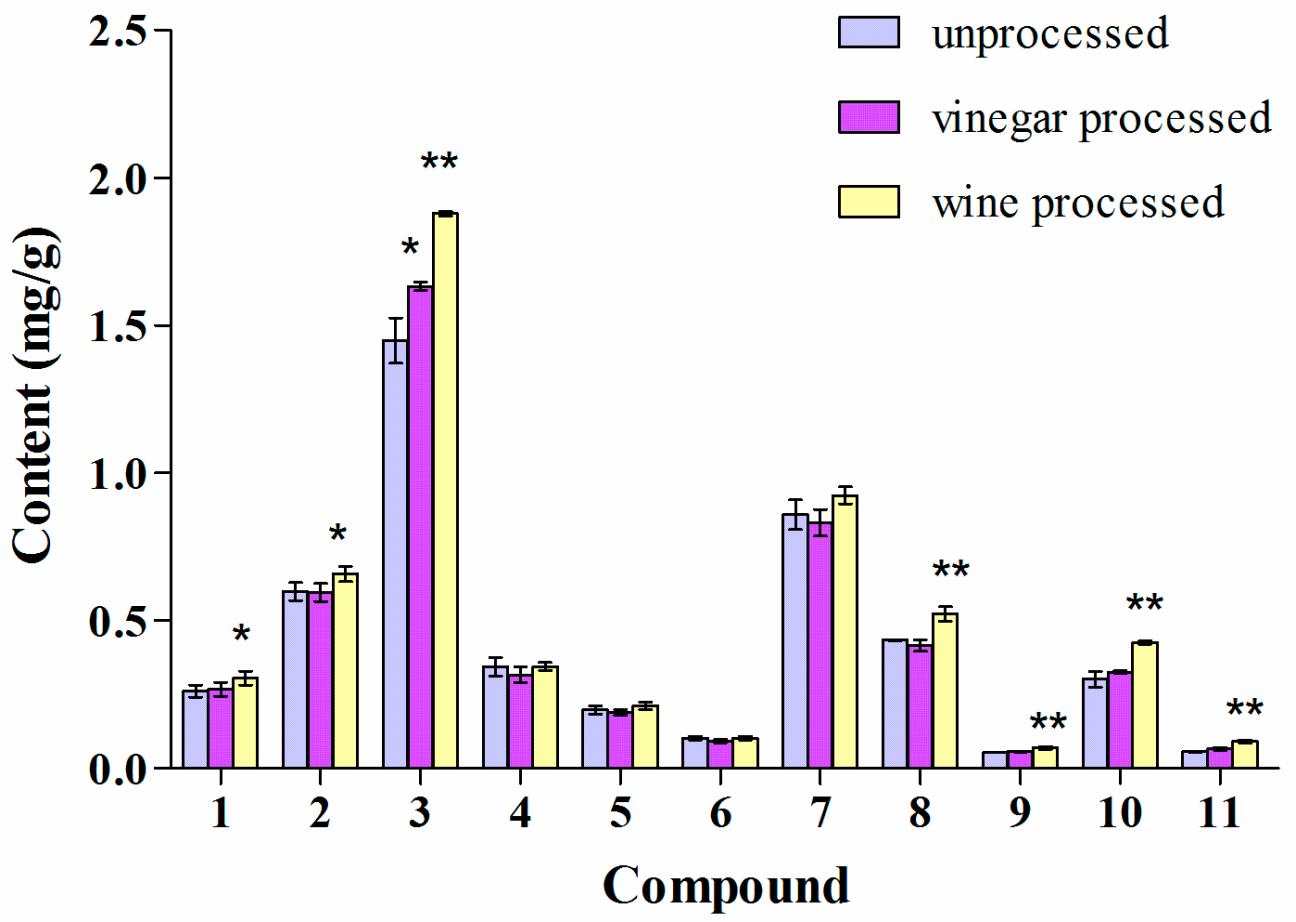
Alkaloid content of the water decoction of JLZS. Values are mean ± SD, n = 3, RSD < 10%. ** *p* < 0.01, * *p* < 0.05 of processed against unprocessed Corydalis Rhizoma.

Previous studies of the water decoction of Yanhusuo reported that vinegar processing can improve the solubility of **1** and **8** [27]. In our study only the amount of compounds **8** and **10** was significantly higher in the water decoction after vinegar processing, while the content of compound **1** was increased by wine processing (Figure 5). The results for the water decoction of JLZS indicated that the contents of all tertiary alkaloids, **1**, **2**, **3**, **8**, **9**, **10**, and **11** were significantly increased by wine processing (Figure 6). Vinegar processing led to a significant increase only for compound **3**.

Previously, improved solubility of alkaloids from Corydalis Rhizoma in the water decoction has been observed after processing. During stir-frying in vinegar, acetic acid can interact with alkaloids, which leads to the formation of water-soluble salts [28]. Yellow rice wine contains phenolic acids [29] that could interact in the same way with alkaloids from Yanhusuo thus, increasing their solubility.

Vinegar and wine processing increased the amount of tertiary alkaloids in the water decoctions, with more pronounced differences for JLZS. In addition, a higher amount of alkaloids was extracted in JLZS when compared to the Corydalis Rhizoma water decoction even when using unprocessed material. This indicated that the solubility of alkaloids in the water decoction can be influenced by the presence of Toosendan Fructus next to Corydalis Rhizoma. The traditional Chinese medicine practice of co-extraction of two or more herbal drugs has been previously shown to increase the concentration of active substances in joint decoctions [30]. A recent example is that of the formula Danggui Buxue Tang which consists of Astragali Radix and Angelicae Sinensis Radix. Ferulic acid from the composition of Angelicae Sinensis Radix, itself an inactive compound, was shown to increase the solubility of active substances from Astragali Radix during the process of co-decoction [31]. It is known that molecules of plant origin such as amino acids, organic acids and sugars can improve the extraction yield and solubility of other compounds [32]. 

Although changes in the alkaloid content were present, the relative content of each alkaloid in unprocessed and processed Corydalis Rhizoma was similar. The two most abundant alkaloids were tetrahydrocolumbamine (**3**) and dehydrocorydaline (**7**). Together, the two alkaloids accounted for 46%–53% of the total alkaloid content in the three analyzed extracts.

According to the Chinese Pharmacopoeia, the quality marker for Corydalis Rhizoma is tetrahydropalmatine (**8**) [1] and our study showed that the amount of the compound was increased by processing. Wine processing elevated the content of tetrahydropalmatine (**8**) in the water decoction of JLZS, while vinegar processing increased its content in the water decoction of Corydalis Rhizoma. Tetrahydropalmatine (**8**) is one of the main alkaloids responsible for the pharmacological properties of Yanhusuo which include analgesic, anxiolytic and cardiovascular effects [33,34,35].

The results also pointed out that in addition to tetrahydropalmatine (**8**) the content of other alkaloids was also increased by processing. For example, vinegar processing caused an elevation of the amount of tetrahydrocolumbamine (**3**) in JLZS and tetrahydropalmatine (**8**) and corydaline (**10**) in the water decoction of Corydalis Rhizoma. Tetrahydropalmatine (**8**) and corydaline (**10**) have been reported to promote a strong antinociceptive effect through the blockade of the D_2_ dopaminergic receptors in the brain [36]. In addition, tetrahydrocolumbamine (**3**) was shown to be a ligand of the dopamine receptor, which plays a role in the perception of pain [37]. Vinegar processed Yanhusuo showed the best analgesic effect in clinical trials and has been used mainly for the treatment of gynecological disorders with particular applications in dysmenorrhea [17]. Hence, the traditional use of the vinegar processed product is in line with the observed increase in the amount of alkaloids with analgesic activity.

According to Bensky *et al.* [17], processing with wine was traditionally believed to contribute to the efficacy of Yanhusuo. Wine processing caused an increase in all tertiary alkaloids [protopine (**1**), α-allocryptopine (**2**), tetrahydrocolumbamine (**3**), tetrahydropalmatine (**8**), tetrahydroberberine (**9**), corydaline (**10**), tetrahydrocoptisine (**11**)] in the water decoction of JLZS. These compounds reported bioactivities such as antinociceptive, antithrombotic, antifibrillatory and antiinflammatory effects [38,39,40]. Hence, the claimed positive influence of wine processing on the properties of the drug could be justified.

## 3. Experimental Section

### 3.1. Chemicals and Reagents

The reference compounds protopine (**1**), α-allocryptopine (**2**), tetrahydrocolumbamine (**3**), coptisine (**4**), palmatine (**5**), berberine (**6**), DL-tetrahydropalmatine (**8**), tetrahydroberberine (**9**), corydaline (**10**), and tetrahydrocoptisine (**11**), were purchased from the National Institute for the Control of Pharmaceutical and Biological Products (Beijing, China) and Chengdu Herbpurify Co. (Chengdu, China). Dehydrocorydalin (**7**) was purchased from Cfm Oskar Tropitzsch GmbH (Marktredwitz, Germany). The purity of the compounds was assessed to be over 95% by reverse-phase HPLC.

Standard stock solutions were prepared by dissolving reference compounds in methanol. A volume of 10 μL was injected into HPLC for analysis. Acetonitrile of HPLC grade was from VWR International (Vienna, Austria). Distilled water was prepared in the laboratory. Triethylamine was supplied by Sigma-Aldrich (St. Louis, MO, USA). Other chemicals, including acetic acid and methanol were of analytical grade (VWR International, Vienna, Austria).

### 3.2. Plant Material

Tubers of *C. yanhusuo* W. T. Wang were purchased from Dongjiaying Country, Hanzhong City, Shanxi Province, China. Dried rhizomes were washed clean, immersed in water until softened, sliced into pieces and dried at 25 °C (“unprocessed Corydalis Rhizoma”). 

### 3.3. Processing of Corydalis Rhizoma According to the Pharmacopoeia [1]

Dried slices were mixed with rice vinegar (20 mL vinegar per 100 g rhizome) or yellow rice wine (20 mL wine per 100 g rhizome) and the slices allowed to completely absorb the vinegar or wine. The soaked slices were then stir-fried over a low flame until they were completely dry (“vinegar processed Corydalis Rhizoma”; “wine processed Corydalis Rhizoma”).

### 3.4. Extraction

For the HPLC analysis all extractions were carried out in triplicate. Prior to analysis, all extract solutions were centrifuged for 30 min at 13,000 rpm.

#### 3.4.1. Methanol Extract

Prepared slices of Corydalis Rhizoma were powdered to a homogeneous size by milling and sieving through No. 3 sieve [1]. Five grams of powder were extracted 3 times with 100 mL methanol for 60 min by hot reflux extraction in a water bath. After filtration, the extracts were combined and concentrated by vacuum evaporation at 40 °C to a volume of 25 mL. A 10 μL volume was injected into the HPLC.

#### 3.4.2. Water Decoction

Ten grams of prepared Corydalis Rhizoma slices were soaked in water (100 mL) for 60 min and then boiled slightly for a further 40 min. After cooling and filtering, the slices were boiled for a second time in water (100 mL) for 25 min. The two decoctions were combined, and the extract was dried by lyophilization. Finally, the extract was dissolved in water and adjusted to a volume of 25 mL. A 10 μL volume was injected into the HPLC.

#### 3.4.3. JLZS Decoction

Ten grams of prepared Corydalis Rhizoma slices and ten grams of prepared Fructus Meliae Toosendan slices were soaked in 200 mL water for 60 min and then boiled slightly for a further 40 min. After cooling and filtering, the slices were boiled for a second time in 200 mL water for 25 min. The two decoctions were combined and dried by lyophilization. Finally, the extract was dissolved in water and adjusted to a constant volume of 25 mL. A 10 μL volume was injected into the HPLC.

### 3.5. Quantitative Analysis of the Alkaloid Content of Corydalis Rhizoma and Its Formulations by HPLC-DAD

Quantitative analysis was performed using a Shimadzu HPLC system (Shimadzu, Kyoto, Japan) equipped with an LC-10AD VP binary pump, an SPD-M10A VP diode array detector, an SCL-10A VP system controller, an auto-sampler, and a DGU-14A degasser.

Chromatographic separation, modified from Ding *et al.* [16], was carried out on a Phenomenex (250 × 4.6 mm, Gemini 5 μm, C18, 110A, SN: 289246-5). The mobile phase consisted of A (0.2% aqueous acetic acid, adjusted with triethylamine to pH 5.0) and B (acetonitrile), using a gradient elution of 20% B holding for 8 min, 20%–36% B within 8–23 min, 36%–80% B within 23–40 min, 80%–95% B within 40–41 min, and 95%–95% B within 41–45 min. The re-equilibration time of the gradient elution was 10 min. The column temperature was 25 °C. The flow rate was 1.0 mL/min. Alkaloids were detected at 280 nm. The alkaloid content for each alkaloid was calculated by comparing the peak area with the standard curve. The total alkaloid content was calculated as the sum of the content of the 11 individual alkaloids.

The method validation was conducted under the above described conditions. The validation included calibration curves, limits of detection, precision, repeatability and accuracy. The stock solution of the mixture of the 11 reference compounds was prepared by dissolving the standards in methanol in a 10 mL volumetric flask. The range of concentrations of each compound in the stock solution was 0.26–0.66 mg/mL. The stock solution was further diluted to the desired concentration. The calibration curve was established with at least six appropriate concentrations measured in triplicate. The limits of detection (LOD) and quantification (LOQ) were determined with the corresponding standard solution at a signal-to-noise (S/N) ratio of ~3 and ~10, respectively.

The intra- and inter-day precisions were determined by analyzing the standard solution containing the 11 marker compounds, with three repetitions daily over three consecutive days. To confirm the repeatability, six different working solutions prepared from the same sample were analyzed. The RSD was taken as a measure of precision and repeatability. The accuracy of the method was estimated by means of recovery experiments with all 11 alkaloids. The recovery was determined by spiking a selected sample. First, the contents of the 11 analytes in the sample were calculated according to their respective calibration curves, before spiking six sample aliquots with identical amounts of the reference compound mixture. Then, the thus fortified samples were extracted and analyzed as described above. The average recoveries were estimated using the formula: recovery (%) = (amount found − original amount) × 100%/amount spiked. 

### 3.6. Qualitative Analysis of Typical Samples by LC-MS

To confirm the presence of the 11 alkaloids in the samples, the mixed standards and typical samples were analyzed by LC-MS. These analyses were carried out using a HCT quadrupole ion trap mass spectrometer (Bruker Daltonics, Bremen, Germany) connected to an UltiMate 3000 RSLC-series system (Dionex, Germering, Germany). As triethylamine has a persistent memory effect [41], the buffer was changed to 10 mM ammonium acetate (adjusted to pH 5.0 with acetic acid), while the other HPLC conditions were the same as those described in Section 3.4. After passing the DAD, the eluate flow was split 1:4 before the ESI ion source, which was operated as follows: capillary voltage: −3.7 kV, nebulizer: 26 psi (N_2_), dry gas flow: 9 L/min (N_2_), and dry temperature: 340 °C. MS^n^ spectra (*n* = 2–4) were obtained in an automated data-dependent acquisition mode (collision gas: He, isolation window: 4 Th, fragmentation amplitude: 1.0 V).

### 3.7. Statistical Analysis

All experiments were carried out in triplicate. Data were expressed as the mean value ± SD. Data analysis between unprocessed and processed Corydalis Rhizoma groups were conducted by two-way analysis of the *t*-test using Microsoft Office Excel 2003. The alkaloid content of each sample was expressed as mg/g of Corydalis Rhizoma dry weight. 

## 4. Conclusions

The present study shows that wine and vinegar processing of Corydalis Rhizoma cause significant changes, not only to the content of the quality marker tetrahydropalmatine, but also of ten other main alkaloids that show pharmacological effects. Processing can increase the amount of alkaloids in the two common formulations used in clinics, namely the water decoctions of Corydalis Rhizoma and its formula Jin Ling Zi San. The differences between the use of processed and unprocessed Yanhusuo are more pronounced for Jin Ling Zi San, in which case the content of all of the studied tertiary alkaloids is increased by processing.
